# Affinity for, and localization of, PEG-functionalized silica nanoparticles to sites of damage in an ex vivo spinal cord injury model

**DOI:** 10.1186/1754-1611-6-18

**Published:** 2012-09-14

**Authors:** Bojun Chen, Mahvash Zuberi, Richard Ben Borgens, Youngnam Cho

**Affiliations:** 1 Center for Paralysis Research, Department of Basic Medical Sciences, School of Veterinary Medicine, Purdue University, West Lafayette, IN 47907, USA; 2 Weldon School of Biomedical Engineering, Purdue University, West Lafayette, IN 47907, USA; 3 Molecular Imaging and Therapy Branch, National Cancer Center, 323 Ilsan-ro, Goyang-si, Gyeonggi-do 410-769, Korea

## Abstract

**Background:**

Traumatic spinal cord injury **(**SCI) leads to serious neurological and functional deficits through a chain of pathophysiological events. At the molecular level, progressive damage is initially revealed by collapse of plasma membrane organization and integrity produced by breaches. Consequently, the loss of its role as a semi-permeable barrier that generally mediates the regulation and transport of ions and molecules eventually results in cell death. In previous studies, we have demonstrated the functional recovery of compromised plasma membranes can be induced by the application of the hydrophilic polymer polyethylene glycol (PEG) after both spinal and brain trauma in adult rats and guinea pigs. Additionally, efforts have been directed towards a nanoparticle-based PEG application.

The *in vivo* and *ex vivo* applications of PEG-decorated silica nanoparticles following CNS injury were able to effectively and efficiently enhance resealing of damaged cell membranes.

**Results:**

The possibility for selectivity of tetramethyl rhodamine-dextran (TMR) dye-doped, PEG-functionalized silica nanoparticles (TMR-PSiNPs) to damaged spinal cord was evaluated using an ex vivo model of guinea pig SCI. Crushed and nearby undamaged spinal cord tissues exhibited an obvious difference in both the imbibement and accumulation of the TMR-PSiNPs, revealing selective labeling of compression-injured tissues.

**Conclusions:**

These data show that appropriately functionalized nanoparticles can be an efficient means to both 1.) carry drugs, and 2.) apply membrane repair agents where they are needed in focally damaged nervous tissue.

## Background

Traumatic CNS injury causes sometimes fatal and often severe neurological and functional deficits. Breaches formed as a result of mechanical damage to the cell membrane readily triggers a series of adverse events compromising the plasma membrane’s essential role, progressive disruption of nearby neuronal tissues, to eventual disruption-induced cell and tissue necrosis [1-5]. As promising strategies for circumventing or controlling such membrane disruption, innovative molecular - or cellular**-**based - therapeutic approaches are now emerging that shows the potential towards clarifying clinical applications [6-8]. Among them, considerable effort has been directed toward the discovery and the administration of synthetic polymers, particularly polyethylene glycol (PEG) or poloxamer 188 (P188), with a hope to promote the enhancement of functional recovery and regeneration [9-17]. Such water-soluble synthetic polymers are capable of effectively restoring the barrier function of damaged membrane and experimental rescue of tissues and cells in a wide variety of model systems. We list a few of both seminal and contemporary examples of these wide ranging investigations: a) rescue of lethally shocked human fibroblasts in vitro using P 188 [17]; recovery from testicular reperfusion injury using the surfactant T-1100 [18]; functional and physiological recovery subsequent to IV injection of the tri-block polymer P188 in guinea pig models of SCI [19]; Restitution of permeabalized skeletal muscle [16]; interruption in the decline in force-induced deficits in contraction in a dystrophic mouse model using P 188 [20]; functional recovery and organelle reassembly in an SCI model using co-polymer – based micro particles [21].

Furthermore, we have shown that parenteral PEG application (Topical, Intravenous, Intraperitoneal, and Subcutaneous) all significantly benefit physiological, anatomical, and functional outcome measures in adult guinea pig models of SCI [5,22], peripheral nerve injury [23], traumatic brain injury [13,14,24], and in acute veterinary clinical cases of SCI in canines [15]. Others have experimented with the addition of Magnesium salts to the PEG solution in SCI (the magnesium having no effect on outcomes) [25] and found benefit in different types of adult rat SCI behavioral models [26,27].

In spite of these clear benefits, there are substantial obstacles for the development of further human clinical trials. Under certain dosage regimens, the molecular weight (MW) and aqueous concentration of PEG can be toxic [28-31]. Indeed, there is a concern for the safety issue of using low MW PEG, given that toxic complexes might be generated during the dissociation process of ethylene glycol into dimers and monomers [32,33]. Additionally, when given by intravenous routes, increased viscosity of PEG as a consequence of high concentration might cause problem in systemic circulation. From a practical standpoint, such limitations would not allow for sufficient flexibility in preparation and application of PEG, and might restrict the appropriate use of *systemic/intravenous* polymers for maximum efficacy and efficiency. To overcome these problems, we have shifted our research focus toward the development of nanoparticle-based membrane applications systems. In previous studies, PEG-functionalized silica nanoparticles (PSiNPs) demonstrated a capability to seal damaged cells and tissues providing neuroprotection subsequent to spinal cord trauma [34-37]. Specifically, we reported the beneficial effects of PSiNPs after systemic application, including physiological recovery of somatosensory evoked potentials (SSEP) following severe compression of guinea pig spinal cords. PEG-tethered nanoparticles offer several advantages over systemic administration of solutions of these various polymers: i) the large surface areas of nanoparticles permit significantly greater PEG delivery at the particular cell/tissue regions of interest. While the systemic footprint of the polymer would likely be undetectable [37], ii) PEG-conjugated particles diminish the current challenges that could arise from the solution-based administration. Indeed, such nanocarrier systems not only minimize the amount of free drug in solution, but also prevent polymer degradation and inactivation upon administration, [36,38] and iii) the small size of nanoparticles affords their internalization inside cells while evading uptake by phagocytic cells [39]. The latter is particularly important given that rescue of lethally poisoned PC 12 cells with Acrolein requires the internalization of PEG [40], also see Summary Figure 6, page 136 in [41]. Acrolein is a potent natural aldehyde toxin created as an endpoint to LPO following mechanical trauma to cells [42].

**Figure 1:**
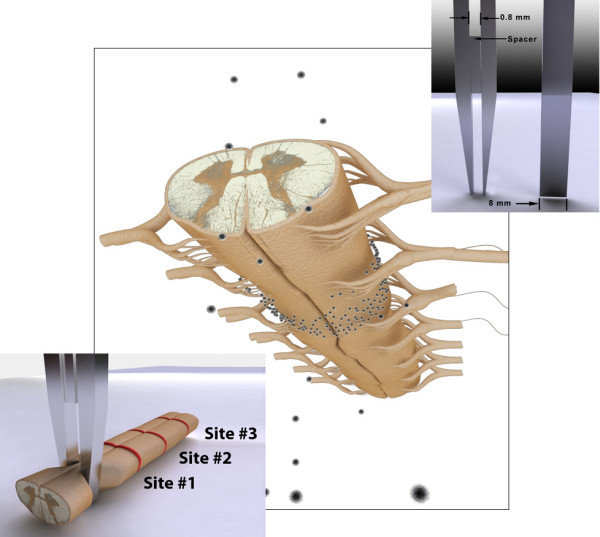
**Schematic illustration of the compression injury to the ex vivo spinal cord tissue.** The bottom left shows the compression injury and adjacent uninjured sites from with 1 cm, 2 cm, and 3 cm gap (site #1, #2, #3). The upper right shows the front and side view of modified forceps with a détente 0.8 mm in width when closed by pressure against the spacer. The isolated spinal cord was placed between the tips of the forceps to induce the compression injury by squeezing for 15 sec against the spacer. The graphic is somewhat exaggerated for the purposes of illustration.

The purpose of this study was to evaluate the selectivity of TMR-doped, PEG-functionalized silica nanoparticles (TMR-PSiNPs) during the repair of damaged spinal cord tissue.

Further understanding of the seminal events following injury-induced CNS cell and tissues damage relative to secondary injury mechanisms will likely provide appropriate and specific treatments for traumatic spinal cord and brain injury, and possibly other neurodegenerative disorders [41,42].

## Results

**Figure 2:**
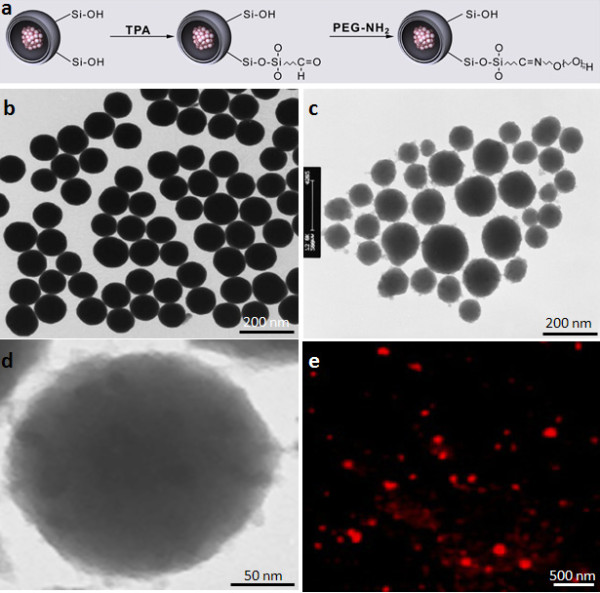
**Decorated Peg-coated Nanoparticles.** (**a**) Diagrammatic synthesis of TMR dye-doped, PEG-tagged silica nanoparticle (TMR-PSiNPs). The particles were separated from microemulsion by vortexing and centrifugating in acetone and ethanol, respectively. (**b**) ~ (**c**) TEM images of bare silica nanoparticles and TMR-doped PEG-coordinated silica nanoparticles (TMR-PSiNPs) with a mean diasmeter of 150 nm. (**d**) shows the magnified image of (**c**) to emphasize the presence of TMR dextran as a black dot in the silica structure. (**e**) Fluorescence microscopic image of TMR-PSiNPs.

The suitable functionalization of the nanoparticles enables a possible preferential recognition of specific cells, facilitating binding and affinity-based endocytosis. Inspired by these important features, silica nanoparticles were functionalized with recognition elements, or PEG, on their surfaces, expecting localization toward particularly injured tissue (Figure 2).

As-synthesized, dye-doped silica nanoparticles with a mean diameter of 150 nm were used for all experiments detailed in this report. The preparation of dye-doped, PEG functionalized silica nanoparticles (TMR-PSiNPs) was initiated via the formation of silica nanoparticles with the entrapment of hydrophilic organic dye molecules (e.g., TMR-dextran) inside the silica pores during hydrolysis and sol–gel polymerization of tetraethyl orthosilicate (TEOS) under the acidic condition (Figure 2(a)). Upon the preliminary particle preparation, surface modification was required to activate the surface with PEG layers by converting the end hydroxyl groups to aldehyde substituents through the reaction with TPA. Consequently, PEG-NH_2_ was conjugated to the aldehyde moiety through Schiff’s base linkage (refer to Figure 2(a)). Figure 2(b) and (c) showed typical TEM images of bare silica nanoparticles and TMR-PSiNPs with mean diameters of 150 nm, respectively. PEG-conjugated nanoparticles showed a flocculant surface morphology, indicating hydrophilic flexible chains of PEG were cross-linked on their surfaces. In addition, the higher magnification image of TMR-PSiNPs emphasizes the presence of TMR-dextran which appeared as black spots (Figure 2(d)). Figure 2(e) shows the fluorescence images of TMR-PSiNPs with optically manipulated fluorescent features, where silica network acts primarily as a supporting barrier to prevent the release of the dye (TMR) from silica pore and to retain its luminophore activity. The chemical composition of modified silica surface was demonstrated by Fourier transform infrared (FT-IR) spectrometer and X-ray photoelectron spectroscopy (XPS), indicating the presence of PEG molecules on the silica surface.

**Figure 3:**
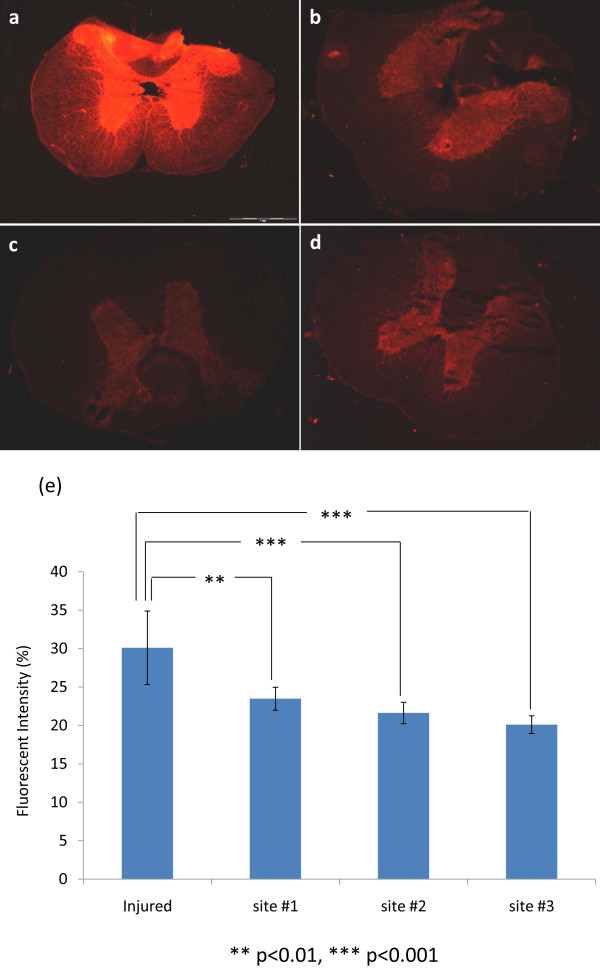
**Localization of Fluorescently decorated Nanoparticles in Spinal Cord.** (**a**) ~ (**d**) The evaluation of injury-specific localization of TMR-PSiNPs to injured and uninjured parenchyma using fluorescence microscopy. Crushed injured vs. uninjured spinal cord tissue are shown by fluorescence microscopy with 50 μm thick frozen sections. (**a**) Compression injured cord section while (**b**) ~ (**d**) uninjured spinal cord tissues with a distance of 1 cm, 2 cm, and 3 cm from crush-injured spinal cord site. Obviously, crushed injury caused intense uptake of large amounts of TMR-PSiNPs represented by significant labeling, especially in gray matter. (**e**) Quantification of fluorescent intensity in injured and uninjured spinal cord with background fluorescence normalized by subtraction of background fluorescence (see methods). Results are expressed as a percent of the uninjured values ± SD (*n* = 5). **P < 0.001, ***P < 0.005.

Meanwhile, to evaluate the selective value of the PEG-conjugated particles, TMR-PSiNPs were tested ex vivo with fifteen isolated spinal cord tissue samples and we assessed the degree of particle accumulation by measuring and quantifying the fluorescence intensity within tissue section as a function of distance from the injured area. Figure 3 displays total fluorescence intensity and distribution of TMR-PSiNPs, as fluorescent features of those particles enable visualization through deep-tissue penetration, particularly within damaged tissues. Interestingly, compression injury induces intense deposition, or localization, of TMR-PSiNPs in close association with lesions where intense labeling in both gray and white matter was observed as shown in Figure 3(a). In contrast to the highly fluorescent labeled injured tissue, tissues in close proximity from the injured area (e.g., site #1, #2, #3) exhibited relatively modest staining, i.e. lower aggregation of particles (Figure 3(b ~ d)). Figure 3(e) showed the quantification of the concentration of TMR-PSiNPs in injured and adjacent to injured (uninjured) tissue with modest distance from the focal injury.

Interestingly, the fluorescent intensity of uninjured tissue sections was about 40% lower than that in the injured tissue. As a control, bare TMR-doped silica nanoparticles (i.e. not coupled to PEG) were employed to observe such adsorption and accumulation within specific injury sites.

**Figure 4:**
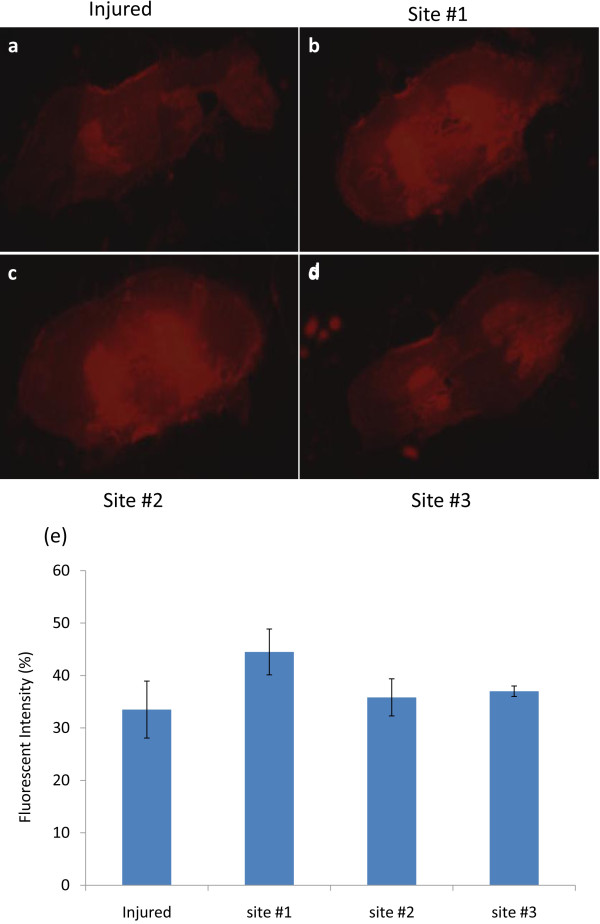
**Nanoparticle Labeling in Injured and Uninjured Spinal Cord.** (**a**) ~ (**d**) The evaluation of accumulation of TMR-SiNPs (without PEG conjugation) to injured and uninjured parenchyma using fluorescence microscopy. (**a**) Compression injured cord section, and (**b**) ~ (**d**) uninjured spinal cord tissues with a distance of 1 cm, 2 cm, and 3 cm from crush-injured spinal cord segment. It was not surprising that specific interaction in the uptake of TMR-SiNPs was not observed when PEG was not a component of the complex. (**e**) Quantification of fluorescent intensity in injured and uninjured spinal cord with background fluorescence normalized and with the subtraction of background fluorescence (see methods). Results are expressed as a percent of the uninjured values ± SD (*n* = 5).

**Figure 5:**
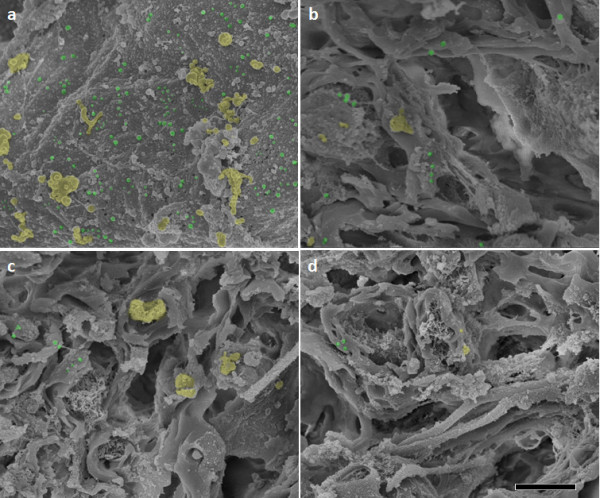
**PSiNP Accumulations visualized by SEM.** The cross sectional SEM images of injured spinal cord (**a**) and uninjured spinal cords located at site #1 (**b**), site #2 (**c**), and site #3 (**d**) upon immediate post treatment with TMR-PSiNPs. Some, but not all, of individual and aggregated nanoparticles have been highlighted in green and yellow. Note the existence of dense particles in Figure 6a.

In Figure 4, a significant difference in fluorescence intensity between injured and normal tissues was not observed. Based on these observations, we suggest that PEG attached to particles is a key factor in promoting anatomical sealing of damaged neuron membranes supported by this affinity. We also qualitatively studied the distribution of nanoparticles using SEM. Given the clear localization to the insult discussed above, an anatomical difference should be apparent. In support of these data, there appeared to be obvious differences in the localization of TMR-PSiNPs along the long axis of the cord with distance from the lesion. The higher-resolution SEM image revealed the localization of the particles within the lesioned tissue whereas the presence of particles was greatly decreased from the injured site as shown in Figure 5.

**Figure 6:**
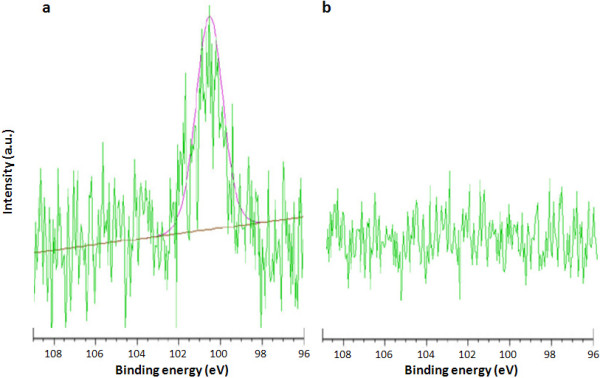
**Evaluating the presence of TMR-PSiNPs in the injured vs. uninjured spinal cords by XPS.** High-resolution XPS surface deconvolution analysis of Si 2p was used to verify the existence of silica nanoparticles on (**a**) crush-injured cord tissue and (**b**) uninjured cord located at site #2. Note that the compression injury increased the level of silica, identifying a precise targeting toward injured regions.

Indeed, the accumulation of nanoparticles to cord tissue was most reduced at site #3, located a distance of 3 cm from the compression injury. Finally, the presence of silica particles within the tissues was also observed using the XPS spectrum (Figure 6). The high**-**resolution Si 2p spectra exhibited an obvious signal through the damaged tissue whereas Si peak was not observed through the intact cord tissues.

## Discussion

### Sealants, Fusogens, and the Damaged Cell Membrane

Since strongly hydrophilic polymers enabled the fusion of intact cells during the formation of hybridomas – it was a short jump in logic to hypothesized that parts of cleaved or diced cells might also be functionally reattached as well. This was first demonstrated by Bittner [43] who cut dissected giant axon from earthworm into two pieces and then subsequently functionally reattached them with PEG. The biophysics permitting the “fusogenic” properties of PEG and multi-block polymers (Poloxamers and Poloxamines) have been investigated for over three decades in cells and model membranes. As mentioned above, a complete understanding of the biophysics and mechanisms of fusion – let alone membrane repair – by these agents still alludes us. A discussion of the biophysics underlying fusion is beyond the scope of this investigative report – but we direct the interested reader to reviews of this subject [4,41,42,44-46].

Once again we provide seminal thoughts from the clear leaders in this scholarship [44,45,47] and contemporary understandings spanning decades to note the evolution in thinking about the mechanisms of action of polymer/surfactant based membrane fusion.

It is agreed that these hydrophilic polymers stabilize closely apposed, yet separate, cell membrane through interaction with, and alteration of, the aqueous phase of membrane amphilic phospholipids, as well as permit or induce the commingling of the hydrophic phase during membrane fusion. As Lee and others have taught – membrane fusion is intimately associated with the organization of water at opposing membranes.

It is difficult to extrapolate experimental and theoretical understandings from polymer – based cell fusion studies to cell repair strategies. In the latter, it is likely that the significant hydrophilia of the repair agent is of prime importance. To rescue damaged but surviving cells, secondary dehydration of the “lesion” may be the most important outcome (refer to summary Figure 2 and 3; page 128 in [41]. In the absence of water within the breech, the lipid phases spontaneously reassemble ie., resolving together as an unbroken phase from the margins. This reestablishes the characteristic form of the membrane and the polar forces associated with the aqueous phase of the reconstituting membrane may reestablish much of the geometry of protein machinery of the membrane held in place in part by these polar forces. These mechanisms, actual and theoretical, probe the ability of hydrophilic polymers to *functionally* reconstitute a variety of cell types and tissues and require more investigation.

### Evaluation of the localization of TMR-PSiNPs in injured and uninjured spinal cord tissues

Recently, we have reported the ability of PEG-coated silica nanoparticles (PSiNPs) in the restoration of integrity of the structurally and physiologically damaged spinal cord tissues [34,37]. However, “targeting” (see below) the molecule to the specific cells/tissues becomes crucial to prevent adverse effects, and to enhance the density of PEG at only sites of damage. This then reduces the actual amount of PEG in systemic circulation at times after administration to vanishingly low.

In this study, crushed cords were incubated in the TMR-PSiNP solution for 15 min followed by the immersion in oxygenated Krebs’ for an additional 30 min. This process might readily account for the reduction in non-specific binding affinity between cord tissues and TMR-PSiNPs, thus indicating low background staining. Overall, our experimental results indicated that total accumulation as an indicative for the presence of TMR-PSiNPs subtly lowered in proportion to the distance from the injured area.

This study model – using the same cord as its own control has been verified and standardized. Briefly, the most sensitive indicator of membrane /tissue damage is an ionic current that flows into the compromised region [48-50]. The magnitude of the current is indicative somewhat of the size of the injury. This is because all cells are inwardly negative and this membrane “battery” drives net current into an insult (be it a region of lowered membrane resistivity or an actual breach). We have measured such currents non- invasively entering axons and tracts of mammalian white matter with vibrating and neutating microprobe systems for the measurement of extracellular current [50]. In these ex vivo mammalian spinal cord segments – injury current is largely restricted to the region of a standardized compression (see below) and falls off steeply by 50 microns from it; see pages 5–6 in Zuberi et al. [50]. The comparative segments of cord are indeed adjacent to the injury zone and provide undamaged white matter for comparison to the central injury. This “control region” of this same cord shows no signs of anatomical or physiological damage. This model we believe is superior to using separate control cords.

We believe that the PEG-conjugated particles specifically provide selective binding to the damaged cell’s surfaces, and thus allows for subsequent intracellular accumulation of significant amounts of nanoparticles with suitably adjusted affinities. In the uninjured segments, there appeared an obvious difference in the labeling of gray and white matter. This is likely due to the fact that neurons large clusters are only found in gray matter. Thus, gray matter may be a more useful site of absorption of particles compared to white matter composed of only nerve axons. This would be an especially important consideration in traumatic brain injury which involves loss of cerebral neurons.

Our data both extends and supports the induction of spontaneous reassembly of damaged membranes of nerves and other cells, their anatomical sealing, and the immediate recovery of nerve conduction through spinal cord by specific localization of PEG where it is most useful.

#### New directions

This paper is a part of a new investigation of several replacement strategies to the application of native PEG for the reasons given above. These also include polysaccharides that are also strongly hydrophilic, can act as “sealants”, are biodegradable, and completely nontoxic [41,51]. Sugars derivatives such as Chitosan may also directly *target* membrane defects using a more appropriated nomenclature.

We [41] as others have used “targeting” to describe a synthetic polymer’s mode of action in repairing cell defects. In reflection this is likely an illegitimate descriptor. It implies a more precise understanding of the mechanisms of specific recognition phenomenon such as observed in ligand / receptor coupling or immunological recognition strategies used in biology and medicine. Thus we have moved away from this term here to describe instead the *affinity* of symmetric polymers for regions of cell and tissue defects that have been empirically reported in SCI and TBI [14]. On the other hand – it may be completely accurate to describe Chitosan’s ability to “recognize” and selectively adhere to cell membrane defects Here the molecular interactions of complex and disorganized lipids and reactive sites on chitosan are understood [41,52,53].

## Conclusions

In this study, we have observed accumulations of TMR-PSiNPs in injured spinal cord tissue compared to nearby “undamaged” tissue. The results revealed a significant interaction of PEG-conjugated nanoparticles toward a compression injury, while there did not appear any apparent association to nearby uninjured tissues. An important fact explaining any aggregation at all in “uninjured tissues” is to recall the amount of handling and surgery used to provide the *ex vivo* samples of spinal cord for study. Indeed, this preferential targeting study strongly supports the initial observations with regard to PEG-mediated spontaneous reorganization, anatomical sealing, and recovery of conduction through guinea pig spinal cord white matter. These data also support PEG – induced dramatic restoration of cerebral cells in damaged brain and recovery of function after such significant injury [24,54,55].

## Methods

### Synthesis and functionalization of TMR-doped, PEG-functionalized Silica

#### Nanoparticles (TMR-PSiNPs)

All chemicals were purchased from Sigma-Aldrich unless otherwise specified. TMR-doped, PEG-modified silica nanoparticles were synthesized according to the previously described procedure [37]. In brief, the mixture composed of 1.77 mL of Triton X-100, 1.8 mL of n-hexanol, 7.5 mL of cyclohexane, and 0.5 mL of 1% aqueous tetramethyl rhodamine-dextran (TMR-dextran, Invitrogen) solution was prepared to form water-in-oil (W/O) reverse microemulsion by adjusting solution pH to 2.0. Then, 100 μL of tetraethyl orthosilicate (TEOS) was slowly added to the mixture with vigorous stirring. The solutions were stirred for another 1 hr until 60 μL of NH_4_OH was added to the solution to terminate polymerization reaction. By adding acetone, the fluffy white precipitate was collected by repetitive vortexing and centrifugation. TMR-doped silica nanoparticles were rinsed with ethanol and acetone several times, and dried under vacuum at 100°C for 12 hr. Subsequently, silica nanoparticles were linked with PEG through two consecutive steps: i) the reaction between silanol groups on silica surfaces and aldehyde groups from 3-(trimethoxysilyl) propyl aldehyde (TPA) and ii) subsequent conjugation of PEG-NH_2_ (M.W. 3000) via a reactive schiff’s base linkage. Particle morphology and size was observed by scanning electron microscope and transmission electron microscope (JEOL 2000FX). The presence of PEG attached on the silica surface was verified using Fourier Transform-Infrared Reflection Absorption Spectroscopy (FT-IRRAS) (data not shown).

#### Isolation of adult guinea pig spinal cords and inducement of injury

All guinea pigs used in this study were handled in accordance with Purdue Animal Care and Use Committee (PACUC) guidelines. Fifteen adult female guinea pigs, of the Hartley strain (300 plus grams) were anesthetized with an intramuscular injection of ketamine (60 mg/kg) and xylazine (10 mg/kg). Then they were perfused through the heart with approximately 500 ml of sterile lactated ringer (SLR) solution to remove the blood. The vertebral column was then rapidly removed and a complete dorsal laminectomy performed along the length of the vertebral column, exposing the spinal cord. The spinal cord was carefully removed from vertebrae and immersed in cold, oxygenated Kreb’s solution (124 mM NaCl, 2 mM KCl, 1.24 mM KH_2_PO_4_, 1.3 mM MgSO_4_, 1.2 mM CaCl_2_, 10 mM glucose, 26 mM NaHCO_3_, and 10 mM ascorbic acid).

A standardized compression injury was induced using a modified forceps possessing a detente by constant mechanical force for 15 sec (Figure 1). Previously reported investigations of compound action potential (CAP) conduction through whole guinea pig spinal cord using the Double Sucrose Gap Isolation / Recording chamber (DSGR**)** verified the immediate and complete loss of conduction through such constant displacement compression injury produced in this way [56,57].

#### Evaluation of targeting ability of TMR-PSiNPs in the spinal cord tissues

As nanoparticle targeting to a desired location improves therapeutic response, TMR-PSiNPs were applied to the segments of ex vivo spinal cord tissues containing compression injury and three different intact sites to evaluate the distribution and localization of the particles. The lesion was centrally located along the long axis of the cord, and the three uninjured sites of study were situated 1 cm, 2 cm, and 3 cm from it (Figure 1). The compression injured spinal cord tissue was incubated in the suspension of TMR-PSiNPs for 15 min followed by the immersion in modified Krebs’s solution for another 30 min. The spinal cords were immediately fixed in 4% paraformaldehyde, sectioned with a freezing microtome with 50 μm thick and evaluated with a fluorescent microscope using excitation/barrier wavelengths of 545/590 nm, respectively. Labeling with a TMR dye was quantified using NIH Image™ software by statistically measuring fluorescence (minus background) intensity. As in prior studies the existence of differing levels of background fluorescence in the samples was normalized by measurement of fluorescence within standardized regions of background at a standard distance from the region of interest – and subtracting this to normalize this data; [37,51].

For Scanning Electron Microscope (SEM) measurements, tissue sections containing particles were fixed in 2.5% glutaraldehyde, and dehydrated with increasing concentrations of ethanol by conventional methods. After drying, the silica nanoparticles were observed after a JEOL JFC-110 ion sputtering (Tokyo, Japan) and analyzed in a JEOL JSM-840 SEM. As another complementary test, the existence of particles within the spinal cord tissues was evaluated using Kratos Axis ULTRA X-ray Photoelectron Spectrometer.

#### Statistical analysis

Unless otherwise specified, unpaired student’s *t* test (for comparison of 2 groups) or one-way ANOVA and Post Hoc Newman Keul’s test (for more than 2 groups) were used for statistical analyses (InStat™ software). Normality was tested by Shapiro-Wilk test (STATA). Equal variances were tested by the method of Barlett for n ≥ 5 (InStat™ software), and by less than 2-fold difference in SD for n < 5. Results are expressed as mean ± SD. P < 0.05 was considered statistically significant.

## Competing interests

The authors declare they have no competing interests. There is no conflict of interest of any sort in the reporting of these data relative to any author.

## Authors' contributions

YC drafted the manuscript and designed the experiments. MZ and BC performed the experiments. RBB is the Principle Investigator and Director of the CPR and is responsible for all elements of the research. All authors read and approved final manuscript.
